# Patients’ Opinions about Knowing Their Risk for Depression and What to Do about It. The PredictD-Qualitative Study

**DOI:** 10.1371/journal.pone.0092008

**Published:** 2014-03-19

**Authors:** Juan Á. Bellón, Patricia Moreno-Peral, Berta Moreno-Küstner, Emma Motrico, José M. Aiarzagüena, Anna Fernández, Carmen Fernández-Alonso, Carmen Montón-Franco, Antonina Rodríguez-Bayón, María Isabel Ballesta-Rodríguez, Ariadne Rüntel-Geidel, Janire Payo-Gordón, Antoni Serrano-Blanco, Bárbara Oliván-Blázquez, Luz Araujo, María del Mar Muñoz-García, Michael King, Irwin Nazareth, Manuel Amezcua

**Affiliations:** 1 Centro de Salud El Palo, Departamento de Medicina Preventiva, Universidad de Málaga, Málaga, Spain; 2 Instituto de Investigación Biomédica de Málaga, Unidad de Investigación del Distrito Sanitario Málaga, Málaga, Spain; 3 Departamento de Personalidad, Evaluación y Tratamiento Psicológico, Universidad de Málaga, Málaga, Spain; 4 Departamento de Psicología Evolutiva y de la Educación, Universidad de Sevilla, Sevilla, Spain; 5 Centro de Salud San Ignacio, Unidad de Investigación de Atención Primaria, Osakidetza, Bilbao, Spain; 6 Centre for Disability Research and Policy, Faculty of Health Sciences, University of Sydney, Sydney, Australia; 7 Servicio de Programas Asistenciales, Gerencia Regional de Salud, Valladolid, Spain; 8 Centro de Salud Casablanca, Instituto Aragonés de Ciencias de la Salud, Departamento de Medicina y Psiquiatría, Universidad de Zaragoza, Zaragoza, Spain; 9 Centro de Salud San José, Linares, Jaén, Spain; 10 Centro de Salud Federico del Castillo, Jaén, Spain; 11 Departamento de Psiquiatría y Medicina legal, Universidad de Granada, Granada, Spain; 12 Unidad de Investigación de Atención Primaria, Osakidetza, Bilbao, Spain; 13 Parc Sanitari Sant Joan de Déu, Fundació Sant Joan de Déu, Barcelona, Spain; 14 Unidad de Investigación de Atención Primaria, Instituto Aragonés de Ciencias de la Salud, Zaragoza, Spain; 15 Department of Mental Health Sciences, University College London, London, United Kingdom; 16 Medical Research Council General Practice Research Framework, London, United Kingdom; 17 Departamento de enfermería, Facultad de Ciencias de la Salud, Universidad de Granada, Granada, Spain; Edinburgh University, United Kingdom

## Abstract

**Background:**

The predictD study developed and validated a risk algorithm for predicting the onset of major depression in primary care. We aimed to explore the opinion of patients about knowing their risk for depression and the values and criteria upon which these opinions are based.

**Methods:**

A maximum variation sample of patients was taken, stratified by city, age, gender, immigrant status, socio-economic status and lifetime depression. The study participants were 52 patients belonging to 13 urban health centres in seven different cities around Spain. Seven Focus Groups (FGs) were given held with primary care patients, one for each of the seven participating cities.

**Results:**

The results showed that patients generally welcomed knowing their risk for depression. Furthermore, in light of available evidence several patients proposed potential changes in their lifestyles to prevent depression. Patients generally preferred to ask their General Practitioners (GPs) for advice, though mental health specialists were also mentioned. They suggested that GPs undertake interventions tailored to each patient, from a “patient-centred” approach, with certain communication skills, and giving advice to help patients cope with the knowledge that they are at risk of becoming depressed.

**Conclusions:**

Patients are pleased to be informed about their risk for depression. We detected certain beliefs, attitudes, values, expectations and behaviour among the patients that were potentially useful for future primary prevention programmes on depression.

## Introduction

Unlike other medical problems such as cancer or cardiovascular disease [1]–[3], very few studies have explored the beliefs and attitudes of the general population concerning the prevention of depression. The first study on this topic involved a national survey of the German public aged 14 years or over [4], which aimed to examine public attitudes towards prevention of depression and public beliefs about helpful preventive measures. Of the whole sample, 75.4% agreed about the possibility of preventing depression, and beliefs about prevention of depression did not conflict with evidence–based programmes. The second and third studies [5], [6] were Australian national surveys of young people. The preventive strategies endorsed by over 80% of young people were: keeping physically active, keeping in regular contact with family and friends, avoiding substances, and making time for relaxing activities [5], [6].

These three studies [4]–[6] enable inferences to be drawn about the populations of their respective countries, though they nevertheless have certain limitations. For example, the exploration of the patients’ beliefs was restricted to the degree of agreement with a closed list of beliefs. Thus, other types of beliefs, attitudes, values, expectations and behaviour remain to be examined and these may be critical for planning and implementing a depression prevention programme.

Our research team conducted the predictD studies [7], [8], which showed that the risk of the onset of a major depressive episode can be quantified in the same way as can other clinical disorders, such as cardiovascular diseases. We now wish to develop primary prevention programmes on depression consisting of the General Practitioner (GP) and the patient sharing information about the risk level (amount of risk) and risk profile (risk factors present) for the patient of suffering major depression (predictD interventions). Before implementing the predictD interventions, we needed to determine the beliefs, values, attitudes, expectations and behaviour about this type of intervention. The development and implementation of any preventive programme may conflict with the perceived needs and expectations of patients [3]. This may jeopardize the implementation of such programmes. This study was undertaken to explore patients’ opinions towards receiving information about their risk for depression and the values and criteria upon which their opinions are based from a qualitative perspective.

## Methods

### Setting

We undertook the study in the primary care setting of seven Spanish cities, from both northern (Barcelona, Bilbao, Zaragoza and Valladolid) and southern Spain (Jaen, Granada and Malaga). Primary Care in Spain is organized in health centres, which each cover a population of 15,000 to 30,000 inhabitants from a geographically defined area. GPs in each health centre work as a group, with extensive primary care teams. The Spanish National Health Service provides free medical cover to 95% of the population. Patients can visit their doctors as often as they want without having to pay for it, even when they do so for preventive reasons. Each patient is assigned to only one GP, who has gatekeeper functions.

### Sample Selection

The study population comprised primary care patients living in one of the seven cities mentioned above. The selection of participants was based on maximal variation to obtain as many perspectives as possible [9]. A maximum variation sample was taken with regard to the criteria of city, age, gender, immigrant status, socio-economic status and lifetime depression (table 1); these features were provided by the GPs.

**Table 1:** Characteristics of the participants in the focus groups.

		Barcelona	Bilbao	Granada	Jaen	Malaga	Valladolid	Zaragoza	Total
N° Patients		8	8	6	10	8	6	6	52
Sex	Male	3	4	4	4	3	3	4	25
	Female	5	4	2	6	5	3	2	27
Age	Young	1	0	3	3	2	3	0	12
	Middle-aged	5	6	1	3	3	2	3	23
	Mature	2	2	2	4	3	1	3	17
Socio-economic level	Low	1	2	1	4	2	2	3	15
	Medium	3	5	3	5	3	2	3	24
	High	4	1	2	1	3	2	0	13
Immigrant	Yes	1	0	0	0	3	1	0	5
	No	7	8	6	10	5	5	6	47
Lifetime depression	Yes	2	4	3	3	4	3	2	21
	No	6	4	3	7	4	3	4	31

Young: 18–34 years; Middle-aged: 35–60 years; Mature: 61–75 years.

The exclusion criteria were an inability to understand and speak Spanish, age younger than 18 or older than 75 years, the presence of psychosis, bipolar disorder or dementia, and suffering, according to their GPs, from major depression at the time of the study. These exclusion criteria are very similar to those of the predictD studies [10], [11].

### Procedure

We used Focus Groups (FGs) with patients as a way of collecting data from several people simultaneously [12], [13], and of examining not only what people think but how they think and why they think that way, since FGs explicitly use group interaction as part of the method [14].

No patient knew any of the other patients participating in their FG. All the participating patients received 20 Euros at the end of the FG as an expression of gratitude for their participation.

The group moderators (facilitators) were experts in qualitative techniques. Moreover, these personnel were independent and not part of the staff of the health centres. Patients were selected and invited to participate by their GPs. Research assistants contacted the patients who were informed of the date, time and place of the meeting. Patients were only told that the purpose of the meeting was to hear their views on various aspects of health to avoid they prepared the topic before focus groups and thus were more spontaneous.

The topic guide used in the FGs consisted of a brief introduction and three general questions, as detailed in Table 2.

**Table 2:** Topic guide for the focus groups.

**a) Introduction to the topic:**
The Predict project has designed, with the help of Spanish primary care patients, a tool to determine the risk for having depression in the next year. We would like to know your opinion about possibly being informed of your particular risk for depression and its possible causes.
**b) General questions:**
From your experience as a patient:
1) Would you like to know this information? (Designed to determine beliefs, values and attitudes)
2) How would you like to be given this information? (Designed to determine beliefs, values and attitudes)
3) If you were given this information right now, what would you do? (Designed to obtain information about attitudes and behaviour)

The FGs were conducted between April and August 2009 and lasted between 60–105 minutes. The FGs were all conducted in quiet areas of health centres and were audio-recorded digitally and transcribed verbatim by the same person (PMP). In the preparation of this manuscript were followed the ‘Consolidated Criteria for Reporting Qualitative Research (COREQ)’ [15] and the ‘Qualitative Research Review Guidelines – RATS’ [16].

### Analysis

We used thematic analysis as suggested by Guest et al. [17]. Using the transcriptions and to ensure data quality, four analysts from the research team (PMP, EM, AF, JP), from different cities and professional backgrounds and blinded to each other, each developed categories based on the responses. Data were segmented by themes, with a mixed generation of categories from the topic guide and those emerging from the data. These themes were identified, coded, re-coded and classified, seeking convergences and divergences.

In order to guarantee the trustworthiness of this research [18], [19], the interviewers and main analysts kept a personal research diary alongside the data collection and analysis to record any reactions to events occurring during the research. In due course, our primary data will be available to other researchers for the purposes of secondary data analysis.

### Ethics Statement

The predictD-qualitative study was approved by the relevant ethics committees in each participating Spanish city: Ethics Committee on Human Research of the University of Granada, Ethics and Research Committee of Primary Health District of Malaga, Ethics Committee for Clinical Research of Sant Joan de Deu Foundation (Barcelona), Ethics Committee for Clinical Research of Aragon (CEICA), Ethics Committee for Health Research of the Jaen Hospital, Ethics Committee for Clinical Research of Euskadi (CEIC-E), Ethics Committee for Clinical Research of the Rio Hortega Hospital of Valladolid. We were aware that patients in the focus groups would be asked to discuss sensitive personal issues with regard to depression (a stigmatized condition) with others in the groups who were relative strangers. Thus, before focus groups were audio-recorded, all participants were informed about the study purpose, its confidentiality and that study findings would be published in academic congress and journals. They could ask questions about it and the group moderators responded to them. They then gave their written informed consent to participate. Participants’ anonymity was kept using personal codes in transcripts.

## Results

The participants comprised 52 patients from 13 urban health centres in seven Spanish cities; 52 of the 70 (74%) patients invited to participate in the FGs consented to take part. The distribution among cities and the characteristics of the patients are shown in Table 1. There was strong agreement between the four independent persons who performed the categorization, both among the persons and regarding the categories designed.

We obtained three main types of response, which were placed within three broad categories: 1) Interest in the intervention, 2) Imparting information, and 3) Consequences for life.

### 1. Interest in the Intervention

Opinion on the possibility of being informed about their risk of depression and possible risk factors.

In general, the respondents had a positive attitude towards knowing their level and risk profile in order to initiate changes in their lifestyle that could prevent the onset of depression, either on their own initiative or originating from the doctor:

[FG Bilbao. Moderator (M): *Well, I’d like you to put yourselves in this situation: before depression starts you go to your health centre and someone tells you that you have a high risk of having depression in the next few months. You haven’t got depression now but you have a risk of getting it and there are a few things that will influence this risk. These are such and such and so and so. What do think about that if it were possible? Patient 13* (P13)*: Very good.* P10*: I think it’s a good idea.* P15*: Anything to do with prevention is great.* P12*: At least you know about it, and then you can start taking precautions*].

Nevertheless, a few patients had doubts about wanting to know the information concerning their risk for depression. The first doubt refers to considering depression a “special” disease: [FG Malaga. P34*: Well, the head’s very difficult isn’t it; if it had been one of those other studies, to do with something else, like cancer, well, then they tell you your blood is thick and you may get it, or your blood is thin or whatever, but the head’s such a strange thing, and you keep on thinking]*. The patients also questioned whether knowing their risk for depression was worthwhile if it failed to help them reduce it: [FG Valladolid. P45*: If it’s something preventive, like a vaccine … for me, yes; but if someone’s going to tell me and then I can’t do anything about it, then no*].

Finally, some patients considered that other persons (not themselves) may be alarmed by the information, with undesired effects: [FG Jaen. P27*: This may pose a problem for weaker persons*]. Less commonly, some responses were negative: [FG Malaga. M*: Imagine someone wants to give you that information.* P35: *Not to me!*]. [FG Valladolid. P45: *I think it’s OK for other diseases, but depression is awful*]. Or responses that were indifferent or sceptic: [FG Malaga. P39: *To tell you the truth, I couldn’t care less.* P37: *It’s all the same, no? I wouldn’t care if they told me, it won’t affect me*].

### 2. Imparting Information

Who should give the information about the risk of depression and how should it be given.

With regard to who is the best person to provide information, patients generally preferred their GP, though mental health specialists were also mentioned: [FG Zaragoza. P50: *the family physician.* P49: *me too, the family physician.* P51: *I also prefer a family physician, a doctor who is a humanist, who knows you, knows what you are like, and who better than your family physician?* P50: *the person who’s been looking after you all your life*].

[FG Zaragoza. M: *And who do you think should be the one to give you that information?* P47: *your family physician is more likely to be correct than a psychiatrist, because they give you medicines that maybe you don’t need and they leave you, or they drug you and you’re asleep all day, or feel groggy; I prefer the family physician rather than a psychiatrist …*]. [FG Valladolid. P43: *these are diseases with an important psychological factor. I think the psychologist…*].

When we asked about the most appropriate way of providing this information, the patients showed a wide and interesting variety of answers:

Receiving the information from the GP but seeking solutions oneself, with or without the GP’s help: [FG Bilbao. P10: *In the end, you look for the solutions yourself. The doctor, well yes, the doctor can tell you you’re going to get depression but you’ll have to deal with it yourself with help*].

Indirectly, using the third person as a more subtle approach, in addition to availability of time for the doctor-patient interview: [FG Granada. P20: *I think it would be good to talk about it a little in general terms, about people who have this and how they get it, they usually have depression, instead of directly, like you’re probably going to get depression; you feel like they’re attacking you, you feel worse; speaking more in general, and secondly, having time*]. Face to face, with visual-facial contact and a coping attitude: [FG Zaragoza. P49: *Me, if I have a disease, I want to be told to my face, and then you know how to cope*]. So that they can understand, simply and directly: [FG Bilbao. P15: *To my face and plainly.* P12: *Naturally*]. Tailored to each patient, bearing in mind the personality of the patient and the negative impact it can have on them, but trying to motivate them: [FG Granada. P22: *Well, it depends. Some people aren’t aware of it, and maybe it’s necessary to put them in a worse situation for them to understand it. Some people are very weak, weak-spirited, and are easily devastated; you have to tell them things very tactfully. It depends on the person, really*].

Involving the family, considering the family resources and the possibility of involving other family members in coping with the risk of depression: [FG Granada. P18: *I reckon I’d need help from my family, ‘cos maybe that sort of information, straight out, may affect you; if you know the husband, or wife, or brother or sister, the family circle, who can tell them, what I have to tell them or how I can tell them; support from the family*].

### 3. Consequences for Life

Behaviour that will be implemented after knowing about the risk of depression.

We found that the responses could be divided into two types:

#### A) What I can do

This category mainly includes strategies related to lifestyle and carrying out an “active life”, in the sense of outside activities and relating with people: [FG Malaga. P39: *Getting out, meeting people, going window-shopping, not sticking at home because that’s awful; it just makes you old and destroys you. What do I do? I get out, go for walks, enjoy myself, I’ve got friends … talk about things with people you trust*]. A few specific activities were also mentioned, like physical exercise, diet, spending time doing hobbies, going out and walking around with people: [FG Jaen. P28: *It’s a good idea to do some exercise, have a balanced diet; you can set yourself a set of rules so that when the problem actually arrives it seems milder.* P27: *a little training in your life, looking at life differently; I’m at greater risk so I’m not going to get bored, I’m going to do sports, going to get about all day*.*].*

[FG Malaga: P34: *everybody who’s got a hobby like sowing, reading, mainly physical; I think that’s better, use up your energy, the depression gets better. Me, when I feel bad and have to go to the doctor, he tells me, get out, get out, get out*. P40: *get out and go for a walk*. P34: *go for a walk, but not by yourself, better with others so you can chat*].

Finally, certain cognitive and emotional control strategies were identified: [FG Zaragoza. P49: *Looking at things positively.* P51: *Seeing things positively.* P49: *Being positive…*].

#### B) What to do and how to help me

It should be noted that (some) other patients mentioned keeping off medication, though they would consider taking them, but not without some reservations. [FG Malaga. P34: *Depression; as far as I’m concerned, I don’t like taking medicine, so I’d take as little as possible, better none*]. Many patients would ask their health care professional for some way to prevent the depression. The request, maybe even demand, that they be given solutions: [FG Jaen. P29: *Well, I’d tell the person who told me that they also have to tell me the remedy …*]. [FG Barcelona. P4: *Well, ask the doctor what to do of course; hell, then tell me what to do about that risk and avoid it …*]. Others would think about participating in informal support groups to help them restructure their lives: [FG Barcelona. P6: *I think that with general get-togethers, in groups, as everyone has problems and that’s life! I reckon, like that, speaking, then you’ll learn a little to live day by day, to look at things another way …*]. Seeking support from family and friends: [FG Granada. P18: *For me, it’s obvious; the family.* P17: *Yes, yes, the family.* P20: *Or friends you can trust …*]. Or going to the mental health specialist to learn about coping strategies in difficult situations: [FG Jaen. P24: *Or maybe go to a specialist, like a psychologist for example, and get him to explain a little about how to have a philosophy about life that will help and not have so much risk of having it, reducing the risk or finding out how to react in difficult situations that can get you down; or make you a little stronger psychologically*].

## Discussion

### Main Findings

In general, the patients showed a positive attitude to receiving information about their risk of future depression. They suggested interesting ideas about how to share information on their risk level and risk profile. They also proposed various different but appropriate attitudes and behaviour to decrease the risk of depression, some of which included asking their GP for advice. This study enabled us to identify proposals and indications to help develop the predictD interventions with the patients’ acceptance, as well as to determine a few barriers and precautions to be taken into account. As far as we are aware, this is the first study to consider patients’ opinions on primary prevention programmes of depression based on their understanding of their risk for depression.

### Strengths and Limitations

One of the strengths of this study is the great variability of the sample, which is a result of the selection criteria used and the cultural variability found in the different regions of Spain included in the study. Our results mirror some aspects (a positive attitude towards prevention of depression and the beliefs about certain preventive behaviour) of the three studies (all quantitative) so far carried out related to this topic, in Germany [4] and Australia [5], [6]. Thus, the findings of our study might be considered in planning prevention programmes for major depression in primary care.

The research team was multidisciplinary (anthropologist, psychologists, GPs, nurses, psychiatrists, social worker, and educational psychologist), the approach was oriented (study methods), and the data were analyzed and interpreted from different professional focuses. In addition, the triangulation between the four analysts for the elaboration of categories was optimal.

Nonetheless, a number of limitations should be mentioned. The first is that the opinions of the patients reflected in this study are based on a potential future predictD intervention and not on a post-intervention evaluation. These results, therefore, will need to be confirmed or rejected after the intervention is applied in practice.

No attempt was made at “respondent validation” as part of a process of error reduction to establish the level of correspondence between researcher and research subjects. Concerning the presence of depression at the time of the study as an exclusion criterion, this was defined by the GP and may, therefore be liable to diagnostic error [20]. Likewise, the selection criterion for lifetime depression, also determined by the GP, might suffer from the same bias.

When the patients were asked about who was the most suitable person to give them information about the risk of depression, the responses may have been slightly skewed towards the GP, as the interviews were conducted in health centres and the study was designed in the context of primary care. However, the persons who carried out the interviews or who chaired the FGs did not belong to the staff of the respective health centres and only one was a GP.

Our wish as researchers was that the patients should favour the establishment of the predictD interventions. This may have influenced by the FGs (interviewer bias) as well as the analysis and interpretation of the data. However, even though most of the opinions of the patients were favourable towards knowing their risk for depression, the analysis of the dissenting opinions was still relevant.

### Comparison with Existing Literature

Several health behavior theories have been used to inform health intervention designs, such as the ‘Theory of Planned Behavior’ [21], the ‘Transtheoretical Model’ [22], the ‘Social Learning Theory’ and the ‘Health Belief Model’ (HBM) [23]. The HBM, developed in the 1950s to investigate why people fail to undertake preventive health measures, although critiqued [24], remains one of the most widely employed [25]. These health behavior models and theories are important because interventions that are informed by theories and models tend to be more successful than those based on intuition [26]. Our data showed a special link between a positive attitude towards knowing their level and risk profile (perception of susceptibility) and the likelihood of initiating changes in their lifestyle that could prevent the onset of depression (perception of benefit). Several patients perceived depression as a different and severe illness (perception of severity), perhaps influenced by previous personal experiences or those of someone close. Some patient related the perceived severity of depression with the desire not to know the risk of depression, which could be linked to stigma, a lower perception of benefit in changing their lifestyle, a greater difficulty (or cost) to perform such behaviors (perception of barriers), or/and simply to a lower perception of self-efficacy. A confidence in one’s ability to undertake health behaviors to prevent depression was an important underlying dimension included in our data; however, other patients showed the opposite, asking their GPs for some way to prevent depression.

The favourable attitude of the patients to primary prevention of depression coincides with other studies [4]–[6], though in our study this positive attitude referred specifically to knowing their level and profile of risk for depression. This is a differential fact that has been unknown up to now.

Certain aspects relating to those patients who were less positive about knowing their risk of depression deserve consideration. The first is the need to have the patient’s consent to receive the information about the risk for depression, both from the ethical point of view and for the safety of the patient. There were suggestions that the information be adapted to the personal and clinical characteristics of each patient, and that professionals communicating the risk should be sufficiently skilled. Thus, from the practical viewpoint, before starting to communicate the risk of depression, the professionals involved should undergo training in both communication aspects (such as giving the information) and in factors related to patient-centred medicine [27], [28]. Participants were aware of the anxiety and the other undesired effects that communicating risk could engender. As with any other preventive activity, specific evaluation is required using clinical trials to measure the frequency of the undesired effect, its magnitude and its importance. Once this is known, the benefit of any potential intervention (depressions avoided) should be determined, together with its secondary effects such as health, quality of life, and cost [29]. Interventions to prevent depression appear effective, although their effects are small to moderate [30]–[31]. However, no trial applies universal prevention (involving patients at low, moderate and high risk) in adults. In our study, the GP was regarded as the most suitable person to provide information on the level and risk profile of major depression. Another study of patients’ beliefs about treatment of depression [32] reported that over the last ten years there has been a reduction in the “deal with it alone” strategy (internal locus control) and an increased perception of the usefulness of health care professionals, e.g., psychologist, counsellor, psychiatrist, social worker [external (professional) locus control]. Over these years the GP has continued to be seen as the most useful person. Another study also found that the GP was the most appropriate person to deal with depression initially [33]. Nonetheless, it should be remembered that these studies refer to the treatment of depression, not its prevention.

Various studies have shown that patients believe psychological and social interventions are more effective than pharmacotherapy for the treatment of depression [34], [35]. In another study, of the 37 proposed actions to prevent depression, psychotropic medication was rated the least helpful (only 6% of people chose it as such) [4]. In our study, the main idea was the desire of the patients not to take any medicines for the primary prevention of depression; though they could accept the possibility of taking them under certain conditions. The use of psychoactive drugs (benzodiazepines and antidepressives) is far higher in Spain than in other European countries [36], which could partly explain the above-mentioned attitude.

The participants suggested a number of attitudes, values, and behaviour to help prevent depression, including undertaking pleasing activities, cognitive restructuring, emotional control, improving social and family support, and physical exercise. Similar results have been found in other studies [4]–[6], coinciding with strategies that have been tested over recent years in different trials on the primary prevention of depression [37]–[41]. These results suggest that empowering the patient may be an important strategy for the primary prevention of depression, introducing the need for prevention interventions that go beyond the limits of traditional face-to-face interventions [42]. Such primary prevention strategies for depression might also be more effective in low-risk populations [43], possibly because this population would be more able to generate, interiorize and set up the resources needed to prevent depression.

### Implications for Future Research and Clinical Practice

Even though patients may have a favourable attitude to knowing their risk, GPs’ attitudes towards the intervention may present barriers to its implementation. Indeed, this situation has already been seen both with other prevention programmes [1], [44], [45] and with the development of psychosocial interventions for the management of patients with medically unexplained symptoms [46] and depression in primary care [47]. ‘Lack of time to do’ was the main barrier to implementing preventive activities that GPs said, and when they were asked about possible alternatives, one of the most frequently proposed was the delegation of these activities in other primary care professionals [1], [44], [45]. Anyway, given that primary care professionals might be responsible for informing their patients about the level and profile of risk for depression, we need to know their opinions about primary prevention programmes for depression based on risk ascertained using the predictD algorithm.

This study provides key information on which to build, and later evaluate, interventions that reduce the incidence of a costly and disabling condition, such as depression. A summary of the key information is shown in table 3.

**Table 3:** Practical implications to bear in mind when designing a primary prevention programme for depression based on the patients’ understanding of their level and profile of risk.

• In theory, according to the patients, the General Practitioner (GP) would be the most acceptable main professional for the primary prevention programs of depression.
• Before offering the interventions seek to understand whether or not the patient wishes to receive such information and respect their wishes at all times.
• The interventions to be developed should facilitate the patient’s ability to change his/her attitudes and behaviour to reduce the risk of depression.
• The interventions should be tailored and given individually, considering the personal, family, cultural and clinical characteristics of each patient.
• The interventions will necessitate the GP having certain interview and communication skills, which will require training.
• The interventions will also require that the GPs have a “patient-centred” approach and attitude, considering the viewpoint and agenda of the patient and involving the patient as a participant in the decision-taking process.
• The interventions might need to include advice to help patients cope with the knowledge that they are at risk of becoming depressed.
• The interventions should also use patients’ internal resources, the family’s resources and the community’s resources: e.g. informal support groups and other resources in society that may be useful to prevent depression (physical exercise, social relations, pleasing activities, etc.).
