# Correction: Patients’ Opinions about Knowing Their Risk for Depression and What to Do about It. The PredictD-Qualitative Study

**DOI:** 10.1371/journal.pone.0101266

**Published:** 2014-06-19

**Authors:** The PLOS ONE Staff

There is an error in the spelling of the eleventh author’s name. The correct name is: Ariadne Runte-Geidel.
